# Supplementary material for: Insect infestations and the persistence and functioning of oak-pine mixedwood forests in the mid-Atlantic region, USA
Source: PLoS One. 2022 May 4;17(5):e0265955. doi: 10.1371/journal.pone.0265955 (PMC9067937; doi:10.1371/journal.pone.0265955)
Supplement: S1 Table — Data are net primary production estimated from USFS Forest Inventory and Analysis data (FIA, [9]) and forest inventory plots in the Pinelands National Reserve (PNR, [13, 30]), simulated net primary production using PnET CN, a process-based forest productivity model [30, 31], WxBGC, a second process-based forest productivity model based on BiomeBGC [32], and LANDIS II, a plot-based model that simulates forest composition, succession, disturbance and other ecological processes linked to the CENTURY succession extension (ver. 3) [33]. Estimated net ecosystem productivity is derived from FIA data, simulated using WxBCG and LANDIS II, and calculated from carbon flux measurements in the PNR [13, 30]. (PDF) [file pone.0265955.s001.pdf]

S1 Table. Productivity of undisturbed oak-dominated, mixed oak-pine, and pine-dominated forests in the mid-Atlantic region. Data are net primary production estimated from USFS Forest Inventory and Analysis data (FIA, [9]) and forest inventory plots in the Pinelands National Reserve (PNR, [13,30]), simulated net primary production using PnET CN, a process-based forest productivity model [30,31], WxBGC, a second process-based forest productivity model based on BiomeBGC [32], and LANDIS II, a plot-based model that simulates forest composition, succession, disturbance and other ecological processes linked to the CENTURY succession extension (ver. 3) [33]. Estimated net ecosystem productivity is derived from FIA data, simulated using WxBCG and LANDIS II, and calculated from carbon flux measurements in the PNR [13,30].

**Variable/Data source**	**Forest type**		
	**Oak-dominated**	**Mixed oak-pine**	**Pine-dominated**
**Net primary productivity (NPP; T C ha^{-1} yr^{-1})**			
FIA data [9]	4.6 ± 0.5	3.8 ± 0.6	4.2 ± 0.5
PNR biometric plots [13,30]	4.5 ± 0.4	3.9 ± 1.1	4.4 ± 0.3
PnET CN simulations [31,32]	5.0 ± 0.5	3.6 ± 0.4	4.3 ± 0.3
WxBGC simulations [33]	5.0	4.8	4.4
LANDIS II simulations [34]	---	4.3 ± 0.3	---
**Net ecosystem productivity (NEP; T C ha^{-1} yr^{-1})**			
FIA data [9]	1.7 to 2.1	1.2 to 1.7	1.0 to 1.6
WxBGC simulations [33]	2.0 ± 0.2	2.1 ± 0.4	2.3 ± 0.6
LANDIS II simulations [34]	---	1.4 ± 0.2	---
PNR carbon flux data [13,30]	1.8 ± 0.3	1.4	1.8 ± 0.3
