# Supplementary material for: Insect infestations and the persistence and functioning of oak-pine mixedwood forests in the mid-Atlantic region, USA
Source: PLoS One. 2022 May 4;17(5):e0265955. doi: 10.1371/journal.pone.0265955 (PMC9067937; doi:10.1371/journal.pone.0265955)
Supplement: S2 Table — Data are presented for the beginning of the study in 2005 before infestation by gypsy moth, and at the end of the study in 2018. Values are means ± 1 SE. Significance levels were tested using ANOVAs and Tukey’s HSD tests, and values indicated with different superscripts among stands are significantly different. (PDF) [file pone.0265955.s002.pdf]

S2 Table. Structural characteristics of the canopy and understory in oak, mixed, and pine stands. Data are presented for the beginning of the study in 2005 before infestation by gypsy moth, and at the end of the study in 2018. Values are means ± 1 SE. Significance levels were tested using ANOVAs and Tukey's HSD tests, and values indicated with different superscripts among stands are significantly different.

**Variable**	**Oak**	**Mixed**	**Pine**	**Statistics**
**Before gypsy moth defoliation in 2005**				
Canopy height (m)	13.9 ± 1.5^{a}	10.6 ± 0.9^{b}	9.7 ± 0.5^{b}	$F = 11.2, P < 0.01$
**Aboveground biomass (tons ha^{-1})**				
Oak trees and saplings	63.6 ± 7.4^{a}	32.3 ± 22.9^{ab}	0.5 ± 0.2^{b}	$F = 5.2, P < 0.05$
Pine trees and saplings	21.3 ± 11.8	19.6 ± 6.1	49.6 ± 10.2	$F = 3.0, P < 0.08$
Total trees	83.1 ± 9.6^{a}	47.6 ± 11.4^{b}	32.7 ± 7.5^{b}	$F = 7.2, P < 0.01$
Total trees and saplings	84.9 ± 12.2	51.9 ± 18.6	50.1 ± 10.2	F = 1.9, NS
Understory vegetation	1.9 ± 0.3^{a}	3.3 ± 0.7^{ab}	4.7 ± 0.8^{b}	$F = 4.8, P < 0.05$
**Following gypsy moth defoliation in 2018**				
Canopy height (m)	13.2 ± 0.3	12.5 ± 1.0	11.1 ± 0.3	F = 2.9, NS
**Aboveground biomass (tons ha^{-1})**				
Oak trees and saplings	52.7 ± 12.7	40.4 ± 29.7	1.1 ± 0.4	F = 2.1, NS
Pine trees and saplings	25.7 ± 7.9^{a}	35.9 ± 10.3^{a}	83.2 ± 11.3^{b}	$F = 9.5, P < 0.01$
Total trees and saplings	78.4 ± 6.8	76.2 ± 21.4	84.3 ± 11.2	F = 0.1, NS
Understory vegetation	3.4 ± 0.4	3.9 ± 0.7	5.0 ± 0.7	F = 1.8, NS
