# Supplementary material for: Insect infestations and the persistence and functioning of oak-pine mixedwood forests in the mid-Atlantic region, USA
Source: PLoS One. 2022 May 4;17(5):e0265955. doi: 10.1371/journal.pone.0265955 (PMC9067937; doi:10.1371/journal.pone.0265955)
Supplement: S3 Table — Values are means ± 1 SE. Significance levels were tested using paired sample T-tests, and values indicated with different superscripts among areas are significantly different. (PDF) [file pone.0265955.s003.pdf]

S3 Table. Structural characteristics of the canopy and understory in uninfested areas and areas infested by southern pine beetle. Values are means ± 1 SE. Significance levels were tested using paired sample T-tests, and values indicated with different superscripts among areas are significantly different.

Variable	Uninfested areas	Infested areas	Statistics
**Canopy**			
Height (m)	15.2 ± 0.3[a]	10.1 ± 1.0[b]	T_{18} = 4.88, P < 0.01
Cover (%)	61.9 ± 2.4[a]	30.8 ± 6.0[b]	T_{18} = 4.81, P < 0.01
**Aboveground pine biomass (tons ha^{-1})**			
Trees	74.2 ± 4.2[a]	2.6 ± 0.9[b]	T_{18} = 16.67, P < 0.01
Saplings	4.0 ± 0.8[a]	3.9 ± 1.3[a]	T_{18} = 0.07, NS
**Understory**			
Height (m)	0.7 ± 0.1	0.6 ± 0.1	T_{18} = 0.71, NS
Cover (%)	71.6 ± 4.6	71.3 ± 8.3	T_{18} = 0.03, NS
