# Supplementary material for: Insect infestations and the persistence and functioning of oak-pine mixedwood forests in the mid-Atlantic region, USA
Source: PLoS One. 2022 May 4;17(5):e0265955. doi: 10.1371/journal.pone.0265955 (PMC9067937; doi:10.1371/journal.pone.0265955)
Supplement: S4 Table — (PDF) [file pone.0265955.s004.pdf]

S4 Table. Meteorological sensors and eddy covariance equipment used to measure turbulence, net ecosystem exchange of CO_2 (NEE) and evapotranspiration (Et) at the oak, mixed and pine stands.

**Variable**	**Instrument/sensor**	**Vendor/model**	**Height(s)**
Turbulence	Sonic anemometer	R.M. Young 81000v,<br>Gill Windmaster Pro	19.5 m or 16 m
CO_2 and H_20	Infrared gas analyzer	LiCor 7000	19.5 m or 16 m
Solar radiation	LiCor 200	LiCor 200	19.5 m or 16 m, 2 m
PAR	LiCor 190	LiCor 190	19.5 m or 16 m, 2 m
Net radiation	NRLite, CNR1	Kipp and Zonen	19.5 m or 16 m, 2m
Air temperature	HMP45c	Vaisala	18.5 m or 15 m, 2 m
Relative humidity	HMP45c	Vaisala	18.5 m or 15 m, 2 m
Precipitation	TE525	Texas Electronics	18.0 m or 15 m
Wind speed	05013	R.M. Young	19.5 m or 15 m, 2m
Wind direction	05013	R.M. Young	19.5 m or 15 m, 2m
Soil temperature	CS-107	Campbell Scientific	5 cm depth
Soil heat flux	HFT-3.1	REBS, Inc.	10 cm depth
