# Supplementary material for: Insect infestations and the persistence and functioning of oak-pine mixedwood forests in the mid-Atlantic region, USA
Source: PLoS One. 2022 May 4;17(5):e0265955. doi: 10.1371/journal.pone.0265955 (PMC9067937; doi:10.1371/journal.pone.0265955)
Supplement: Attachment — Submitted filename: PONE-D-21-34003 review.docx [file pone.0265955.s005.docx]

This paper looks at successional changes in forests due to outbreaks of SPB and *L. dispar*. It appears as though there are a couple different objectives, but those are never clearly stated in the manuscript, so it doesn’t really become clear what the paper is about until the discussion. There is a lot going on in this paper and, at times, can get confusing to read. The objective in the abstract is to “understand ecological consequences of invasive insects on…..” but that’s pretty vague. Also, you discuss southern pine beetle which is not an invasive insect under some/many definitions. Overall, the paper leaves out some major details in terms of objectives and methods. I believe the authors did a massive amount of work on this and simply need to be more specific and intentional in their writing. My other main issue is with consistency in writing and explanations.

General comments:

- Change all instances of “gypsy moth” to *Lymantria dispar* as the common name is being changed.
- The authors define phrases/words many times throughout the paper while still continuing to spell them out. At the same time, some things are stated but never defined. For instance, ecosystem water use efficiency is define at least 3 times while the authors don’t use the acronym WUE.

Introduction:

- The intro feels a bit out of order.
- L43: Add commas around “…and intensity”
- Remove L52-54.
- L70-71: This sentence implies that the authors are going to discuss vulnerable species somewhere but this doesn’t come up throughout the paper.
- L92: add “primary” to NPP definition
- L114: This whole paragraph could be much earlier, I think.
- L122: The authors have not defined NEE yet.
- L137: Define and replace “course woody debris” with CWD
- L146: Remove “…as summarized in the conceptual model in Fig 1” and simply cite (Fig 1) at the end of the sentence.
- L147-148: Sounds like this is what the authors are setting up to investigate but this is different than the “objective” in the abstract.
- L153-155: Define these as acronyms and then consistently use the acronyms throughout.
- L245: the authors use N for nitrogen here but in other places it is spelled out. Be consistent.

Methods:

- There are SDs for precip but not temp. Don’t think SD are needed at all in site descriptions.
- The sections on *L. dispar* and SPB all seems like intro material.
- L222: If plots were set up in a 4 x 4 arrangement, how could there be 10-16 plots? Should always be 16….
- L226: Capitalize DBH and use throughout. Don’t need 1.27 m as DBH already has a definition. Height should have (m) after it to show how you measured. How was crown condition assigned? There is no description of crown condition anywhere.
- L230: Why is recruitment in here twice?
- L231: What is a clip plot?
- L251-252: Did you extract data from these papers and then add them to your analyses?
- L254: use [N] instead of spelling out “concentration” each time. Also, is content different than concentration? Again, these things are not well defined.
- L267: Why use SD instead of SE? Also, this seems like a huge SD!
- L274: How was cover measured? This is not clear.
- L277: I’m confused as to what fuels have to do with anything. Fuels (like for fires?) have not been brought up at all yet.
- L285: NEE should be defined earlier and should be the strict definition (i.e., net ecosystem exchange)
- L315: How did you assume it was dry? Were there certain environmental variables you checked beforehand? If so, then it’s not really an assumption per se.
- L321: First time basal area is mentioned. Should be defined as BA with BA being used throughout the rest of the paper.
- L332-334: This should be the first thing in the stats section
- L323: Software used should be at the end (assuming you used that software for all analyses)
- L330: How many subsets?

Results

- L339-347: Results are very vague (i.e., “….and CWD were low at all three stands…”). What is “low”?
- L372: “Stem increment”?
- L378-379: Are these values ± SD or SE?
- L382: Again, results are vague with “very low” values and no means or other descriptors that let us know what “very low” actually means.
- L400-407: These types of results are not given for *L. dispar*. This is more what I expect in a results section.
- L426: What is “course wood mass”? Do you mean CWD?
- L430-435: Seems like an intro sentence to the discussion.

Discussion

- L505-507: Can you generalize this to “insect infestations”? You only looked at one herbivore and one bark beetle…
- L509: This is the first time that “vulnerable” has come up again. By vulnerable, do you mean listed or simply susceptible to herbivory? Not really sure what vulnerable means in the context of this paper.
- Were there any areas that had BOTH SPB and *L. dispar*?
- L519: First time fire management has been mentioned. Is this related to “fuels” that came up earlier? Or unrelated? Fire really isn’t mentioned elsewhere.
- L533: Are you using N content as a proxy for “foliage quality”? If so, this isn’t defined.
- L599: Add end parentheses to the end of the sentence
- L602-605: Wildfires are brought up here but, again, it’s not clear whether this was something actually looked at by the authors

Tables and Figures:

- Figure 1: Shouldn’t it be “southern pine beetle infestations”? You didn’t look at any other “pine beetles”.
- Table 1: Add “(PNR)” to caption after you define Pinelands National Reserve as the authors use PNR in the table but do not define it.
- Table 2: I don’t know that a column with “figure” is necessary here
- Figure 2 caption: This should stand alone. The authors don’t define “Inf” here.
- Table 3: Again, I don’t think a column for figure is necessary. And, since the authors simply report whether p-values are sig or not, this could be done with an * next to the T value instead and take up considerably less space.
- Table 5: Same comments as above.

Check references. Some are abbreviated while others are not. Some journals are abbreviated while others are not. Make sure you are consistent with journal requirements.
