# Supplementary material for: Insect infestations and the persistence and functioning of oak-pine mixedwood forests in the mid-Atlantic region, USA
Source: PLoS One. 2022 May 4;17(5):e0265955. doi: 10.1371/journal.pone.0265955 (PMC9067937; doi:10.1371/journal.pone.0265955)
Supplement: Attachment — Submitted filename: Response to Reviewers.docx [file pone.0265955.s006.docx]

Dr. Karen Root

Academic Editor

PLOS ONE

Re: PONE-D-21-34003, Insect Infestations and the Persistence and Functioning of Oak-Pine Mixedwood Forests in the Mid-Atlantic Region, USA.

Dear Dr. Root,

We appreciate the opportunity to submit a revised version of our manuscript, entitled “Insect Infestations and the Persistence and Functioning of Oak-Pine Mixedwood Forests in the Mid-Atlantic Region, USA”, with Kenneth L. Clark, Carissa Aoki, Matthew Ayres, John Kabrick, and Michael R. Gallagher as authors.

Your comments and those of the reviewers have been very helpful, and we feel that following this guidance has improved the quality of our manuscript considerably. We first provide an overview of our revisions and changes to the manuscript. We then detail our responses to comments by the Editor and Reviewers below this, with our responses in italics. Finally, we address a number of points that were raised in your letter regarding PLoS One editorial requirements.

Thank you for considering our manuscript for publication in PLoS One.

**Detailed replies to comments by the Editor:**

Your paper addresses the very interesting question of how insect infestations may affect forest composition, carbon dynamics, and hydrological cycling in northeastern forest stands.  This is a particularly important question as damage from pests, such as gypsy moths and pine beetles, seems to be increasing.

While this could be an important contribution to our approach to managing forests, I agree with the reviewers that the paper has a lot of promise but needs some additional strengthening and clarification.  As Reviewer #2 points out, there is a lot of information in the Introduction, but the purpose is not as clear here as in the discussion.  There are some very good recommendations that they make to improve clarity and flow in the Introduction.

*Thank you for the encouraging set of comments. We agree with the Editor’s and Reviewer #2’s assessments of the Introduction section, and have reorganized some of these paragraphs in the revised manuscript. Two paragraphs that were not essential to developing the research questions and objectives have been edited; and 1) the paragraph that discussed “associative resistance” has been removed and integrated with the Discussion section, and 2) the paragraph on the productivity (NPP and NEP) of undisturbed oak-dominated, mixedwood, and pine-dominated stands has been condensed, with the extensive Table 1 moved to Supplemental S1 Table.*

There are a number of places where the descriptions of the different sites surveyed is confusing.  Please note some of the issues highlighted by both reviewers throughout the methods and the results.

*We have condensed and moved the sub-section on “L. dispar and southern pine beetle” in the Materials and Methods section. We had intended this to summarize the impacts and spatial distributions of recent infestations in the region, but it was probably more confusing that helpful. We now only describe the spatial extent of the two recent infestations that we studied in Pinelands National Reserve of New Jersey in the appropriate subsection for forest census measurements.*

*Further, we have reversed the order of presentation of the FIA-type plots sampled pre-, during and post L. dispar infestations for clarity. We have also rewritten the description of the FIA-type plots that were sampled for southern pine beetle infested areas, highlighting that only untreated stands were used in our analyses here (please see comments below).*

In particular, it is difficult to disentangle the labels for the pine sites that are infested or not by southern pine beetle and treated (managed?) or not.  Or is the treatment status ignored for the purposes of your comparisons?

*We first must apologize for a typographic error on the label for pine sites in Figure 4, which from left to right presents the relative basal areas of an oak-dominated stand pre- and post-infestation of L. dispar, a mixed stand at the beginning of the study, and pine-dominated stands post- and then pre-infestation of southern pine beetle, with the changes driven by infestations indicated with arrows. We had inadvertently switched the order of “post-“ and “pre-“ for the pine sites, and have corrected this in the revised version.*

*Although we measured forest structure in stands that were untreated and had been treated for southern pine beetle infestations (treatments included “cut and leave” and “cut and chip”; detailed in an annual report for the USFS Forest Health and Monitoring program in Clark et al. 2017 [24]), we have only used data from the 10 untreated, naturally occurring infestations here. We omitted data from the sites where suppression treatments were conducted because a major difference between untreated and treated stands is that pine saplings were cut or damaged in the treated stands, and this obscured the impacts of southern pine beetle and the shift to stands that more closely resemble mixedwood composition, as shown in Figure 4. We have rewritten our description of this in the Methods section, and now mention the treatment types explicitly in the Methods section.*

Reviewer #2 has provided extensive line by line suggestions that should be carefully addressed.

*Reviewer #2 has provided very helpful line by line comments, and we have attempted to address all of these. We feel following these suggestions has improved the clarity of the manuscript, and we appreciate the time Reviewer #2 spent on reviewing our previous draft.*

The discussion repeats a lot of the results but could be strengthened by discussing the broader context and complicating factors such as fire, climate change, and herbivory and reducing the reiteration of the results.

*Both the Editor and Reviewer #2 suggested that the Discussion section could be improved by first reducing the reiteration of the Results, and then expanding the broader context and complicating factors. In the original version of the manuscript, we intended to summarize the Results in the Discussion section first, noting that we are presenting a complex set of results that has integrated data from long-term forest census plots, FIA-type sampling, and long-term flux data from three sites that have been variously disturbed by insect infestations and prescribed fires. However, we agree with the Editor and Reviewer #2 and have removed the sub-sections headings and condensed the three sub-sections on impacts of L. dispar and southern pine beetle on forest composition, structure and productivity into three paragraphs. Further, we have expanded linkages to the conceptual model in Figure 1 by referencing this where appropriate, as per the comment below.*

Figure 1 is very useful as a framework for the paper and it should be revisited more substantially in the discussion.  For example, there is little discussion of differential impacts in uplands and lowlands but rather an emphasis on pine versus oak.

*In the original version of the manuscript, we largely limited our discussion to upland systems, because all of our research on L. dispar and the three carbon flux towers are located in upland forest stands. While we believe that a more extensive treatment of lowland forests would be interesting, we felt this would be beyond the scope of our analyses. However, we have expanded our references to southern pine beetle effects in lowland systems throughout the revised manuscript. We also have expanded our discussion of fire return intervals and the fact that hardwood tree species in lowland forests, primarily red maple and black gum, are more fire intolerant than oaks in the revised Discussion section.*

I agree with Reviewer #2 that there should be more discussion of fire, especially since fuels were measured (Line 277) for at least some sites. Does fire in these systems both prescribed and wild complicate conclusions about the effects of the infestation and resilience to disturbance?

*We have expanded our discussion of fire, which does appear throughout the original version of the manuscript but was not highlighted particularly well.*

*Your question is an excellent one, and throughout the Discussion of the revised manuscript we have attempted to show that fire, especially the current patterns of extensive use of prescribed fire and wildfire suppression, would tend to reinforce the persistence of uneven age mixedwood stands because it promotes the regeneration of both oaks and pines by reducing understory competition and removing excess litter layer on the forest floor. An abundance of research has been conducted on the effects of low intensity fire in the Pinelands National Reserve and through the mid-Atlantic region that we now cite in the revised manuscript. In addition, we now explicitly cite how the effects of insect infestations and fire are consistent with a recently published conceptual model of mixedwood formation and persistence (Kern et al. 2021 [22]}.*

It would be useful to discuss some of the implications of these findings and how they might apply in other contexts.

*We have attempted to expand our discussion of the implications of our study throughout the revised Discussion section.*

Both reviewers provide some suggestions to improve the tables and figures.  For example, Reviewer #1 suggests more contrast is needed for Figure 6b and should more closely resemble 6a. You might even want to similarly improve the contrast of Figure 5.  The figures are generally helpful but there is no description in the figure legends what the arrows on the graphs are indicating.

*We have used better contrasting colors for Figures 5a and 5b, and 6a and 6b. We have also provided a description of what we intended the arrows to indicate in these two figures. The sentence stating, “Arrows indicate the directional changes in forest structure and composition following L. dispar infestations.” has been added to the legend below Figures 5 and 6.*

This paper has a lot of promise and could be a good addition to the literature with some improvements.

*Thank you again for supporting our manuscript*

**Detailed replies to comments by Reviewer #1:**

Reviewer #1: Great work, just a few minor edits found.

*Thank you.*

Line 42 would be good to note that it is a decrease in sever wildfires. *We have added the phrase “…and a decrease in the occurrence of severe wildfires [1-3].*

Line 59-60 reword, for example: These "mixedwoods" are characterized by neither hardwood nor softwood exceeding 75% dominance. *We have reworded this sentence to read, “These “mixedwoods” are characterized by neither hardwoods or softwoods exceeding approximately 75% dominance [e.g., 19-21].”*

Line 87 says oak pine mixedwoods, but isn't it also making lowland deciduous pine mixedwoods?

*We agree. We believe that Reviewer #1 intended to mean hardwood pine mixedwoods, and so we have added a phrase to mention this. We have also pointed this out explicitly throughout the Discussion section.*

Line 177 you put a ; in 36,654 ha. *The semi-colon is now a comma in 36,654 ha for the acreage of wildfires from 2004 to 2016.*

Line 200-201 would be good to clarify timing by putting year(s) in parenthesis after pre-, during, post-. *Thank you for pointing this out. We have added the years in parenthesis for each of these periods.*

Line 221 why was it 10-16? were some removed due to location issues?

*Yes, some plots fell on paved or sand roads, or in the case of the pine dominated site an unforested fire break. These were either not sampled or omitted from our analyses here. We now state this more clearly in the revised manuscript.*

Line 222 has "(see below)" but doesn't refer to anything, is there supposed to be a Figure?
Tables with sub-sections should have that sub-section header in Bold or Italics to help with reading the table.

*This statement referred to the flux towers, but we agree this was confusing. We now state “(described below)” to clarify.*

*Thanks, this is a helpful comment for table presentation. We have reformatted all of the tables with sub-section headers in bold.*

Figure 6b would look better with more contrasting colors.

*We have increased the contrast in Figures 5 and 6 by lightening the color of the bars indicating water use efficiency values for spring periods.*

**Detailed replies to comments by Reviewer #2:**

This paper looks at successional changes in forests due to outbreaks of SPB and *L. dispar*.

It appears as though there are a couple different objectives, but those are never clearly stated in the manuscript, so it doesn’t really become clear what the paper is about until the discussion.

There is a lot going on in this paper and, at times, can get confusing to read. The objective in the abstract is to “understand ecological consequences of invasive insects on…..” but that’s pretty vague.

Also, you discuss southern pine beetle which is not an invasive insect under some/many definitions.

Overall, the paper leaves out some major details in terms of objectives and methods. I believe the authors did a massive amount of work on this and simply need to be more specific and intentional in their writing. My other main issue is with consistency in writing and explanations.

*These are all very helpful comments. We do agree that this is a complex paper, and have reorganized and rewritten the Introduction section so that our objectives are easier for readers to follow. Following this, we have condensed some of the subheading topics throughout the Methods and Discussion section, for example the “L. dispar and southern pine beetle” section has been condensed into the sections on forest census measurements for L. dispar and southern pine beetle. We have also rewritten much of the Discussion section.*

**General comments:**

Change all instances of “gypsy moth” to *Lymantria dispar* as the common name is being changed. *We have replaced “gypsy moth” with “Lymantria dispar” at first use and “L. dispar” throughout the remainder of the manuscript.*

The authors define phrases/words many times throughout the paper while still continuing to spell them out. At the same time, some things are stated but never defined. For instance, ecosystem water use efficiency is define at least 3 times while the authors don’t use the acronym WUE.

*We apologize for the inconsistencies. We have defined all terms at first use, then use the correct acronyms throughout the revised manuscript.*

Introduction:

The intro feels a bit out of order.

*We agree, and as noted above, we have rewritten the Introduction section so that relevant material is covered in such detail, and the development of our questions and then objectives are clearer.*

L43: Add commas around “…and intensity” *Thank you, this is clearer now.*

Remove L52-54.

*We have substituted this introductory sentence, and now introduce the Pinelands National Reserve here, as Fig 1 (the conceptual model) addresses forests in the PNR.*

L70-71: This sentence implies that the authors are going to discuss vulnerable species somewhere but this doesn’t come up throughout the paper.

*We agree with Reviewer #2 that the use of the term “vulnerable” is vague and could be interpreted as meaning the conservation status of a species. Thus, here and on line 509 we have omitted the term “vulnerable” and have reworded to “susceptibility to insect infestations…”*

L92: add “primary” to NPP definition *OK, now corrected.*

L114: This whole paragraph could be much earlier, I think.

*We agree because this is a key objective and differs from the Abstract. We have rewritten much of the Introduction of the revised manuscript to address this and comments above.*

L122: The authors have not defined NEE yet. *We now define “net ecosystem exchange of CO_2_” before the first use of “NEE”*

L137: Define and replace “course woody debris” with CWD

*We now define course woody debris as CWD on first use and use CWD throughout the remainder of the revised manuscript. We also report values for the oak, mixed and pine stands, and refer to previously published values. Thanks, this was not clear in the last version.*

L146: Remove “…as summarized in the conceptual model in Fig 1” and imply cite (Fig 1) at the end of the sentence. We have shortened this sentence as suggested, thanks.

L147-148: Sounds like this is what the authors are setting up to investigate but this is different than the “objective” in the abstract.

*We have reworded the single objective listed in the Abstract so that it is a better description of actual objectives of our study. We have also revised much of the Introduction section.*

L153-155: Define these as acronyms and then consistently use the acronyms throughout.

*We have now defined all terms and acronyms at first use and used the correct acronyms throughout the manuscript. Again, we apologize for the inconsistencies.*

L245: the authors use N for nitrogen here but in other places it is spelled out. Be consistent.

*We have used the abbreviation “N” for nitrogen throughout the manuscript, except at first use where it is defined, and when it appears at the beginning of a sentence. Thank you, this is clearer now.*

Methods:

There are SDs for precip but not temp. Don’t think SD are needed at all in site descriptions.

*In the revised manuscript, we have followed a standard protocol on reporting means and SD’s for air temperature and precipitation, reporting averages over the last 30 years. We have added SD values to the temperature data presented in the text.*

The sections on *L. dispar* and SPB all seems like intro material.

*We considered moving these two paragraphs to the Introduction, but believe that they are best shortened and included as part of the Materials and methods section. However, we have removed the general information on susceptible species and only reported the years and extents of these infestations in southern New Jersey, under the descriptions of the forest census plots. This is important information to include somewhere, because it does indicate the extent to which L. dispar and SPB have impacted forests in the Pinelands National Reserve.*

L222: If plots were set up in a 4 x 4 arrangement, how could there be 10-16 plots? Should always be 16….

*This is true, but some plots were not sampled or not included in the analyses because one or more of the FIA subplots fell on sand roads or disturbed, non-forested areas. We apologize, this was not written very clearly in the previous version.*

L226: Capitalize DBH and use throughout. Don’t need 1.27 m as DBH already has a definition.

*We have changed all uses of “dbh” to “DBH”.*

Height should have (m) after it to show how you measured. How was crown condition assigned? There is no description of crown condition anywhere.

*We used the standard Forest Inventory and Analysis protocol for assigning crown classes for trees. These are emergent, dominant, co-dominant and suppressed. We do not present those data here, but would include it in the archived data.*

L230: Why is recruitment in here twice? *We apologize for this typographical error. This sentence now reads correctly.*

L231: What is a clip plot?

*These were destructively harvested plots measuring either 1.0 m2 or 0.5 m^2^ used to determine the aboveground biomass of understory vegetation and saplings. We have reworded this sentence in the revised manuscript.*

L251-252: Did you extract data from these papers and then add them to your analyses?

*These cited papers had additional [N] data for growing season foliage of the dominant and co-dominant species that we sampled. We compared our results to theirs, and their averaged values were reasonably close to ours.*

L254: use [N] instead of spelling out “concentration” each time. Also, is content different than concentration? Again, these things are not well defined.

*We now define N concentration as [N] following first use. We also define N content clearly, and use this term throughout the manuscript. Thank you for pointing this out, it is clearer now.*

L267: Why use SD instead of SE? Also, this seems like a huge SD!

*We used the SD value because that was reported by Aoki et al. in the cited publication.*

*These values came from tree ring counts of cored trees in stands sampled by Aoki et al., and their sampling occurred in the same stands we report here (their transects were co-located with the FIA type plots we installed and sampled in some stands).*

L274: How was cover measured? This is not clear.

*Cover was visually estimated from 4 cardinal directions out from the center point of each plot and then averaged for cover of understory vegetation and tree saplings. We have added “visually estimated“ in the text description. We would also include these values in the archived data.*

L277: I’m confused as to what fuels have to do with anything. Fuels (like for fires?) have not been brought up at all yet.

*We do mention wildfires and prescribed fires early in the Introduction, and effects of fire on species composition are also mentioned when describing the conceptual model in Figure 1 in the revised manuscript. We then return to the effects of fire in the Discussion, where we discuss the importance of fire in the regeneration of pines and oaks, and also describe how mixedwood forests may be less prone to severe wildfires compared to pine-dominated forests which have greater amounts of ladder and crown fuels. We have removed the mention of “available fuels” for pine trees and saplings here because we do not report these values in Table S2, which presents structural characteristics of areas infested by southern pine beetle and uninfested areas. Again, we would report these in the archived datasets.*

L285: NEE should be defined earlier and should be the strict definition (i.e., net ecosystem exchange). *We have defined NEE at first use in the revised manuscript.*

L315: How did you assume it was dry? Were there certain environmental variables you checked beforehand? If so, then it’s not really an assumption per se.

*We couldn’t really measure amounts of the water on leaf, needle and other canopy surfaces, so we thought it more accurate to use the term “assumption” here. We followed the protocol in previously published accounts of calculations for water use efficiency, WUEe., and used local half-hourly precipitation data to estimate dry periods.*

L321: First time basal area is mentioned. Should be defined as BA with BA being used throughout the rest of the paper.

*We have now defined “basal area” as BA at the first use, and now use the term BA throughout the remainder of the manuscript.*

L332-334: This should be the first thing in the stats section

*This is a good point. We have moved this sentence from the end of the paragraph to the beginning, because this was tested first before proceeding with the statistical analyses.*

L323: Software used should be at the end (assuming you used that software for all analyses)

*We did use SYSTAT 12 for all of the statistical analyses, and have moved this sentence to the end of the paragraph in the Statistics section.*

L330: How many subsets? We have reorganized this sentence for clarity, and now report this value as “25 subsets”.

Results

L339-347: Results are very vague (i.e., “….and CWD were low at all three stands…”). What is “low”?

*We have chosen to present the results for L. dispar chronologically, following their impact on*

*the three stand types because this highlights their differential impact on forest composition and structure. We now present CWD values throughout the Results section in the revised manuscript. Thank you, this was too vague in the previous version.*

L372: “Stem increment”? *We have substituted the term “BA” for “stem” to be consistent with the use of basal area, and for clarity.*

L378-379: Are these values ± SD or SE? *We have now defined these values above as SE.*

L382: Again, results are vague with “very low” values and no means or other descriptors that let us know what “very low” actually means. *We agree that this is vague and have added values to the text for coarse wood.*

L400-407: These types of results are not given for *L. dispar*. This is more what I expect in a results section.

L426: What is “course wood mass”? Do you mean CWD? *Yes, and we have changed this to CWD throughout the manuscript. This seems clearer now.*

L430-435: Seems like an intro sentence to the discussion.

Discussion

L505-507: Can you generalize this to “insect infestations”? You only looked at one herbivore and one bark beetle…

*This is true, and we have rewritten this sentence in the revised manuscript to indicate that we only investigated the effects of the two forest insects.*

L509: This is the first time that “vulnerable” has come up again. By vulnerable, do you mean listed or simply susceptible to herbivory? Not really sure what vulnerable means in the context of this paper. *We agree, and have omitted the term “vulnerable” throughout the text, because what we intend is “susceptible to herbivory”*

Were there any areas that had BOTH SPB and *L. dispar*?

*This is a good question. We observed stands with patches of oaks which likely had been previously impacted by L. dispar (as indicated by larger dead and damaged oak trees) adjacent to SPB stands that had been treated using cut and leave or cut and chip treatments. We sampled the SBP portions of these stands, but unfortunately because treatments had been conducted in the SBP portions of the stands, we did not use that information here because treatments also reduced the basal area and biomass of pine saplings. Perhaps in the next infestations?*

L519: First time fire management has been mentioned. Is this related to “fuels” that came up earlier? Or unrelated? Fire really isn’t mentioned elsewhere.

*We have attempted to highlight fire throughout the revised manuscript. We have also strengthened the discussion about use of prescribed fires and wildfires in regenerating pine and oaks, and fire-intolerance in some hardwoods. Thank you, this was a very good comment, and we attempted to improve the discussion of fire throughout the revised manuscript.*

L533: Are you using N content as a proxy for “foliage quality”? If so, this isn’t defined.

*Yes, and we now define this in paratheses in this sentence. Thanks for pointing this out, because it makes our intended use of the N content information clearer.*

L599: Add end parentheses to the end of the sentence. *OK, thank you.*

L602-605: Wildfires are brought up here but, again, it’s not clear whether this was something actually looked at by the authors.

*We now provide a number of citations throughout the manuscript that have either investigated the reduced occurrence of wildfires because of suppression activities or simulated the impacts of wildfires on forest composition, structure and ecosystem functioning. We also mention how the pine dominated stand had been burned in a wildfire in 1995, and subsequently in prescribed fires in 2008 and 2013, and that three prescribed fires have been conducted at the mixed pine-oak stand. Further, we have rewritten a paragraph in the Discussion section summarizing the documented the effects of prescribed fire in promoting the regeneration of oaks and pines in the PNR and throughout the mid-Atlantic region. This is important information for understanding the persistence of uneven age mixedwood stands that we did not treat very effectively in the previous version of the manuscript, and we appreciate this set of comments.*

Tables and Figures:

Figure 1: Shouldn’t it be “southern pine beetle infestations”? You didn’t look at any other “pine beetles”.

*Yes, this is true. We have substituted the term “Southern pine beetle infestations” in Figure 1. We have also changed “Gypsy moth” to “Lymantria dispar” for consistency.*

Table 1: Add “(PNR)” to caption after you define Pinelands National Reserve as the authors use PNR in the table but do not define it. *We have added “(PNR)” to the end of the last sentence of the caption for Table 1.*

Table 2: I don’t know that a column with “figure” is necessary here.

*We have left the references to the figures in the Tables because we feel that it will allow readers to find significance levels for statistical tests easily.*

Figure 2 caption: This should stand alone. The authors don’t define “Inf” here.

*We now define Pre, Inf, and Post in the caption for Figure 2, using the following definition:*

*“Pre” indicates before infestations, “Inf” indicates during infestation in 2007, and “Post” indicates a decade following infestations.*

Table 3: Again, I don’t think a column for figure is necessary. And, since the authors simply report whether p-values are sig or not, this could be done with an * next to the T value instead and take up considerably less space. Table 5: Same comments as above.

*Following the comment above, we left the references to the figures in each table. We feel that this will allow readers to find this information more easily.*

Check references. Some are abbreviated while others are not. Some journals are abbreviated while others are not. Make sure you are consistent with journal requirements.

*We have reformatted the references so that they are consistent with PLoS One instructions. We apologize for this error.*

**Editorial comments for PLoS One formatting**

*For Point #1 and the last comment by Reviewer #2, we have paid much closer attention to the proper formatting for PLoS One manuscripts, including the Tables and References.*

2. In your Methods section, please provide additional information regarding the permits you obtained for the work. Please ensure you have included the full name of the authority that approved the field site access and, if no permits were required, a brief statement explaining why.

*We now provide information on how we obtained permission to sample sites for both sets of forest census plots. Nearly all lands were New Jersey state forests or wildlife management areas managed by the New Jersey Department of Environmental Protection.*

3. Please remove any funding-related text from the manuscript and let us know how you would like to update your Funding Statement.

*For Point #3, we have removed the funding-related text from the Acknowledgement section in the revised manuscript. We would like our online funding statement to remain unchanged, and read: “Partial support for this project was provided by USDA Forest Service Forest Health and Monitoring Program grants NE-EM-F-13-01 to KC and NE-EM-B-12-01 to MA and AK.”*

*Thank you for changing this on the online submission form.*

*For Point #4, we would first like to clarify that some of the information in the manuscript has been previously published, and where we have used previously published information we have provided the appropriate citations. In summary, forest census data and carbon flux data from the three sites used to analyze the impacts of L. dispar previous to 2016 have been published in peer-reviewed publications. Forest census and carbon and hydrologic flux data for the “post” period in 2018 are unique to this manuscript. Similarly, summaries of the forest census data from the stands infested by southern pine beetle have been published in an annual report for the USFS Forest Health program, and in a meeting proceedings in 2020. These are also cited where used in this manuscript. Using this information is essential in setting the documenting the pre-infestation conditions for L. dispar and southern pine beetle, and for documenting some of their impacts through time. The overall conceptual model of mixedwood forest formation and persistence, and the analyses of forest productivity and hydrologic data in the context of mixedwoods is unique to this manuscript.*

Thank you again for considering our revised manuscript for publication in PLoS One.

Sincerely,

Kenneth L Clark
